# A Pictorial Essay of Coronary Artery Anomalies on Coronary CT Angiography: A Single-Centre Observational Study

**DOI:** 10.7759/cureus.64398

**Published:** 2024-07-12

**Authors:** Armeen Ahmad, Sudeep Roplekar, Anna Podlasek

**Affiliations:** 1 Radiology, Prime Hospital, Dubai, ARE; 2 Radiological Sciences, University of Nottingham, Nottingham, GBR; 3 Radiology and Imaging Technology, University of Dundee, Dundee, GBR

**Keywords:** cardiac ct, congenital heart diseases, retrospective observational study, ct coronary angiography, coronary artery, cardiac anomaly, congenital cardiac anomaly

## Abstract

Background

Congenital anomalies of the coronary artery anatomy (CAAs) encompass a spectrum of disorders, often asymptomatic but potentially carrying severe clinical implications such as arrhythmia, chest pain, myocardial infarction, or sudden death. The estimated prevalence of CAAs in the general population ranges from 0.3% to 1.3%, with underdiagnosis in asymptomatic individuals. Multidetector computed tomography angiography (CTA) has emerged as a vital non-invasive tool for diagnosing and characterising CAAs, offering improved visualisation and aiding in appropriate management decisions. This study aims to analyse the spectrum of CAAs in a tertiary care setting, focusing on imaging features, prevalence, and potential clinical significance, utilising data from patients who underwent multidetector CTA.

Methodology

A single-centre, retrospective analysis of consecutive coronary angiograms over a five-year period identified patients with CAAs, with imaging conducted using a 128-slice, single-source CT scanner. Detailed imaging evaluation was performed by experienced radiologists, with anomalies classified according to established criteria.

Results

Among 756 coronary CTA examinations analysed, 37 instances of anomalous coronary vessels were identified. The study revealed a diverse range of anomalies, including myocardial bridging, anomalous origin of coronary arteries, and extracardiac abnormalities.

Conclusions

This study contributes valuable insights into the prevalence and imaging features of CAAs, enhancing our understanding of these anomalies and guiding improved patient outcomes in cardiovascular care. Future research should focus on elucidating pathophysiological mechanisms and establishing multicenter registries to address the challenges associated with studying these infrequent but clinically significant anomalies.

## Introduction

Congenital anomalies of the coronary artery anatomy (CAAs) represent a spectrum of congenital disorders of the coronary artery anatomy. The majority represent an asymptomatic nature; yet, symptomatic manifestations, which include arrhythmia, chest pain, syncope, myocardial infarction, or sudden death, underscore their potential for severe clinical implications [1,2]. They should be regarded as variants rather than congenital anomalies when the anatomical features of the coronary arteries are prevalent in more than 1% of the general population [3,4]. The estimated prevalence of CAAs in the general population ranges from 0.3% to 1.3%, but this figure may be underestimated due to underdiagnosis in asymptomatic individuals [5-8]. The complexity of CAAs, including anomalies of origin, course, and termination, poses unique challenges in clinical decision-making.

Multidetector computed tomography angiography (CTA), with its superior spatial resolution and rapid acquisition times, has become an indispensable tool in the non-invasive diagnosis and detailed characterisation of CAAs. Better visualisation aids in the differentiation between benign and potentially hazardous coronary artery configurations, thereby guiding appropriate management strategies [1,9]. Moreover, this provides an opportunity for clinicians to conduct extended follow-ups and assess anomalies functionally to determine their hemodynamic significance. However, its extensive adoption is restricted by the dosage of ionising radiation and the utilisation of contrast agents, especially considering that a majority of patients are young [10].

This study aims to analyse the spectrum of CAAs identified in a tertiary care setting, focusing on their imaging features, prevalence, and potential clinical significance. By examining a comprehensive dataset of patients who underwent multidetector CTA, this research provides valuable insights into the morphological characteristics and variations of CAAs, contributing to improved diagnostic accuracy and patient management strategies.

## Materials and methods

In this single-centre, retrospective study, all consecutive coronary angiograms were reviewed over five years, from January 2018 to December 2023, at a tertiary care facility, to identify the variant of CAAs. This subset of patients who presented with CAAs formed the basis of the study, underscoring the prevalence of these anomalies in a clinical setting.

Imaging protocol

The diagnostic protocol involved using a 128-slice, single-source CT scanner, employing retrospective electrocardiographic gating. This method, combined with administering 75 mL of intravenous contrast material followed by an 80 mL saline chaser and a 15 mL contrast for a test bolus, optimised the visualisation of coronary arteries. Additionally, formal calcium scoring was conducted to assess the extent of coronary calcification, which can influence the management of coronary artery disease.

The imaging protocol was designed to achieve high-resolution images, with section thicknesses of 0.6 mm during breath-hold. Images were reconstructed with a 1 mm overlap to ensure detailed visualisation of coronary artery structures. Volumetric reconstructions were obtained for each patient, allowing for a comprehensive assessment of the anatomy of coronary arteries.

Imaging evaluation

Two experienced radiologists (AA and SR with 20 and 10 years of experience) retrospectively assessed each CTA study to identify the coronary artery’s origin, course, and termination. The decision to classify the study as anomalous with a variant according to the CAA classification (Table 1) was made through consensus. The use of volumetric images, owing to their three-dimensional nature, proved beneficial in illustrating the origin and course of the anomalous coronary artery, as well as its relationship to cardiac chambers and adjacent great vessels. This thorough approach guaranteed the accuracy and reliability of the assessment and classification.

**Table 1:** Simplified nomenclature of coronary artery anomalies. Adapted from [11].

Anomaly group	Anomaly example
Anomalies of origination and course	- Coronary ostium in improper coronary sinus: right coronary artery originating from the left coronary sinus, anterior descending and circumflex arteries originating from the right coronary sinus, with proximal course anomaly (interarterial, retroaortic, prepulmonic, and transseptal)
	- Coronary ostium outside the aortic coronary sinus: pulmonary artery, left ventricle, right ventricle, ascending or transverse aorta, etc.
	- Single coronary artery
	- Absence of the left coronary trunk
Intrinsic anomalies	- Atresia or congenital ostial stenosis, ectasia or aneurysm, hypoplasia or agenesis, etc.
	- Intramural course (myocardial bridge) or subendocardial
	- Split right coronary artery and anterior descending artery, anomalous origin of the posterior descending artery or first septal branch
Termination anomalies	- Inadequate arteriolar/capillary branching
	- Fistulas

The study received approval from the Institutional Review Board (IRB) of Prime Hospital, Dubai (approval number: 01.04.2024) and adhered to the ethical standards and policies of the hospital.

The results are presented as counts. Microsoft Excel® (Microsoft Corp., Redmond, WA, USA) was used for data summary.

## Results

In this single-centre, retrospective study conducted over a five-year period from January 2018 to December 2023, a total of 756 coronary CTA examinations were analysed, revealing 37 instances of anomalous coronary vessels.

Anomalies of origination and course

**Figure 1:**
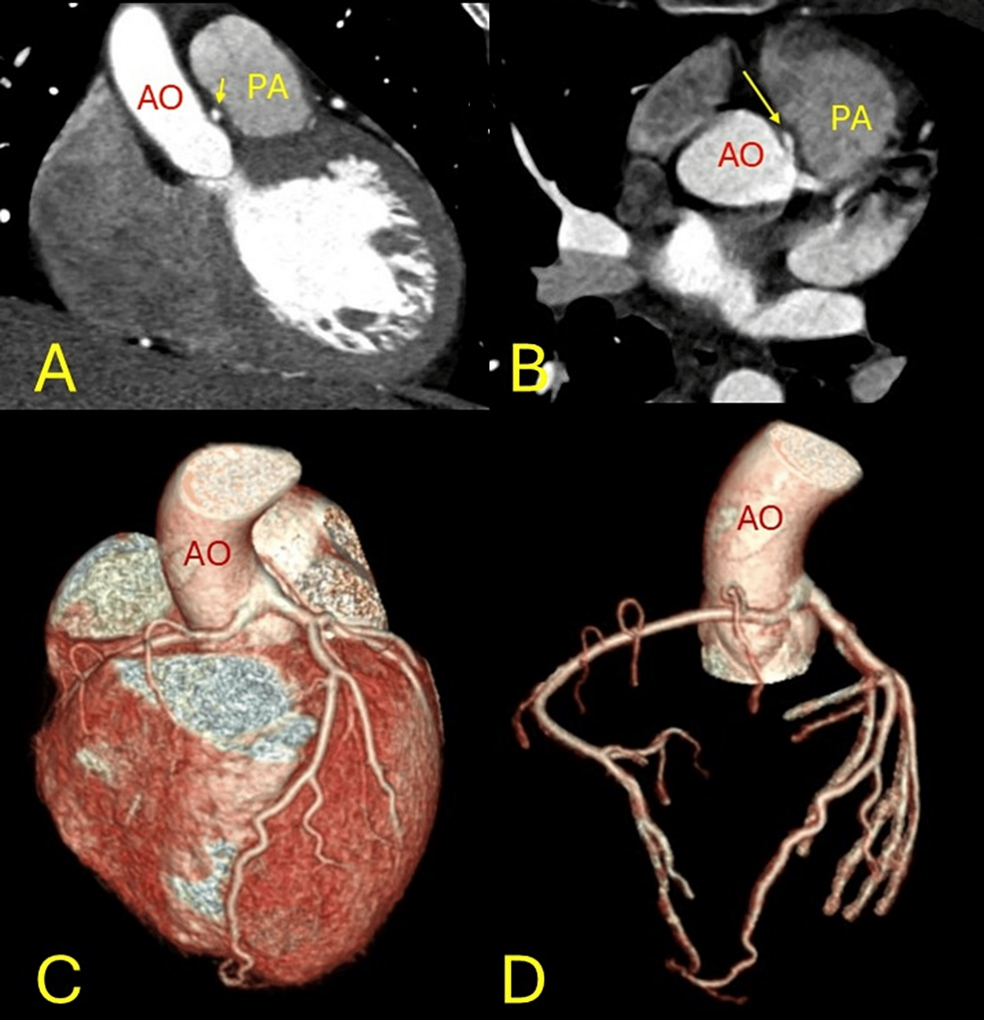
Anomalous origin. Anomalous origin of the right coronary artery from the left coronary sinus (yellow arrows) with malignant interarterial course between the pulmonary artery (PA) and aorta (AO). A: left coronal, B: right axial images. Three-dimensional coloured volume rendered images C: left, D: right.

**Figure 2:**
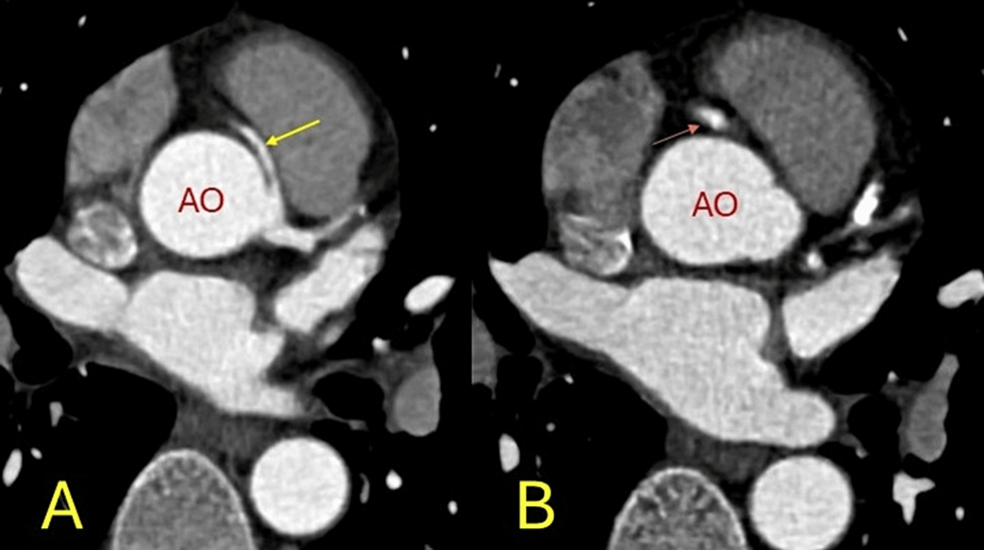
Anomalous origin. The right coronary artery arising from the left coronary sinus (yellow arrow) with a calcified plaque (orange arrow) in its proximal segment. The aorta (AO) is in red. A, B: axial images.

**Figure 3:**
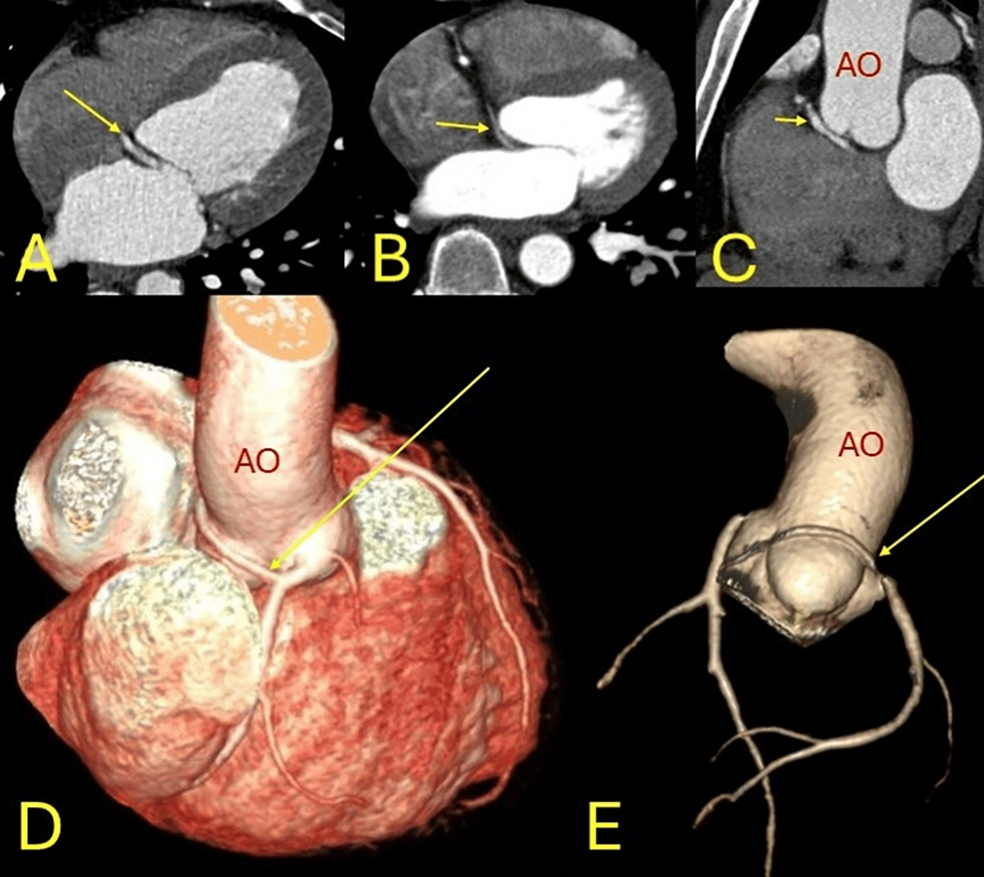
Anomalous origin. The left circumflex coronary artery arising from the right coronary sinus with preaortic course (yellow arrow). AO (aorta in red). A, B: axial, C: coronal reformatted images, D, E: three-dimensional coloured volume rendered images.

**Figure 4:**
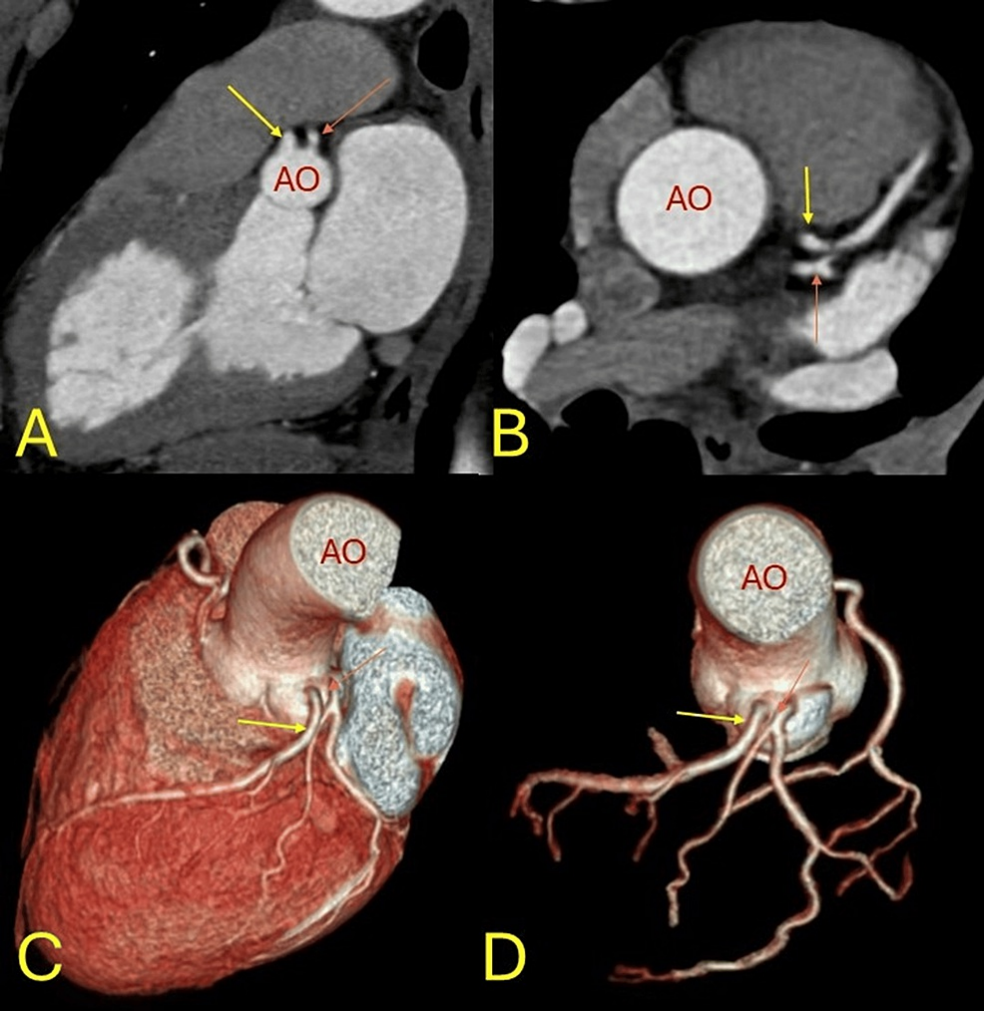
Anomalous origin. Separate ostial origin of the left anterior descending (yellow arrow) and left circumflex (orange arrow) coronary artery from the left side of the aorta (AO) at the level of the coronary sinus with absent left main coronary artery. AO is in red. A: sagittal, B: axial images, C, D: three-dimensional coloured volume rendered images.

**Figure 5:**
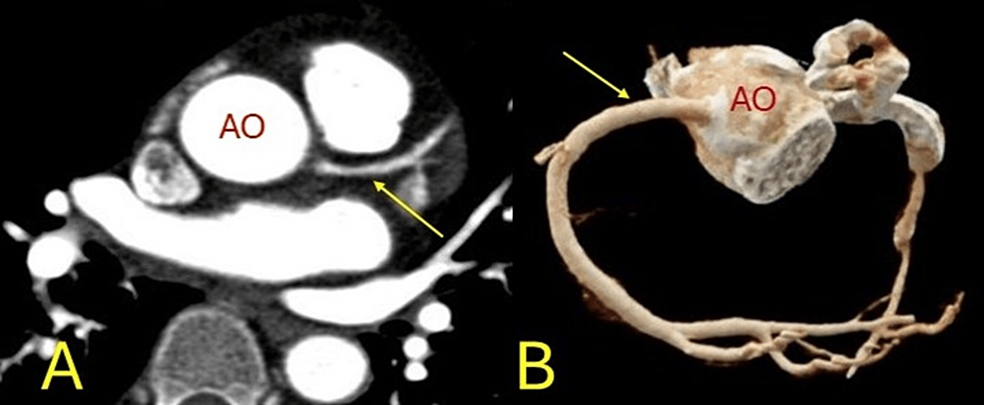
Anomalous origin. Absent left circumflex coronary artery with left main coronary artery continues as the left anterior descending artery (yellow arrow). Aorta (AO in red). A: axial, B: three-dimensional coloured volume rendered image.

We noted nine anomalies of origin and course, including three counts of right coronary artery (RCA) arising from the left coronary sinus with malignant interarterial course (Figures 1, 2), one count of separate origin of the left anterior descending artery (LAD) and circumflex artery (Cx) from the left coronary sinus (Figure 3), four counts of left Cx arising from the right coronary sinus (preaortic course in one and retroaortic in three) (Figure 4), and one count of absent left Cx (Figure 5).

Intrinsic abnormalities

**Figure 6:**
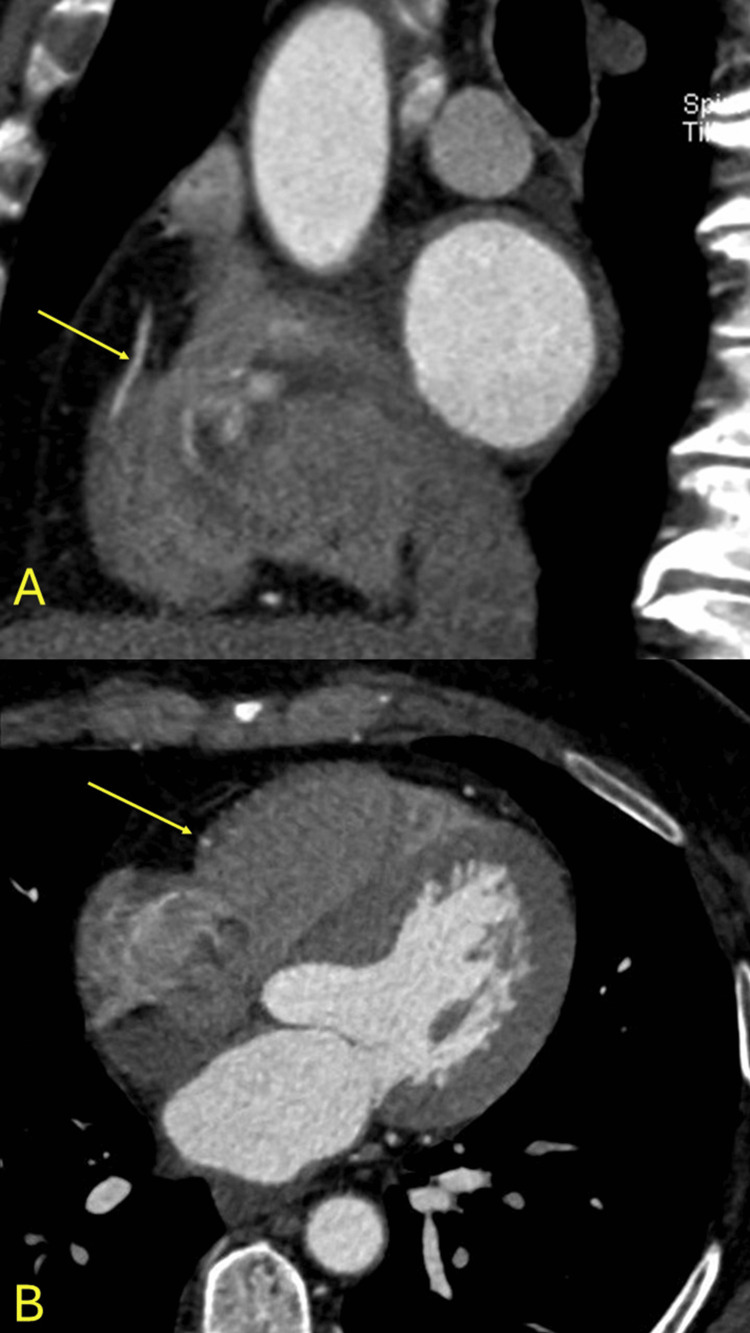
Superficial bridging. Superficial bridging in mid-right coronary artery without luminal stenosis in the bridging segment (yellow arrow). A: oblique sagittal, B: axial images.

**Figure 7:**
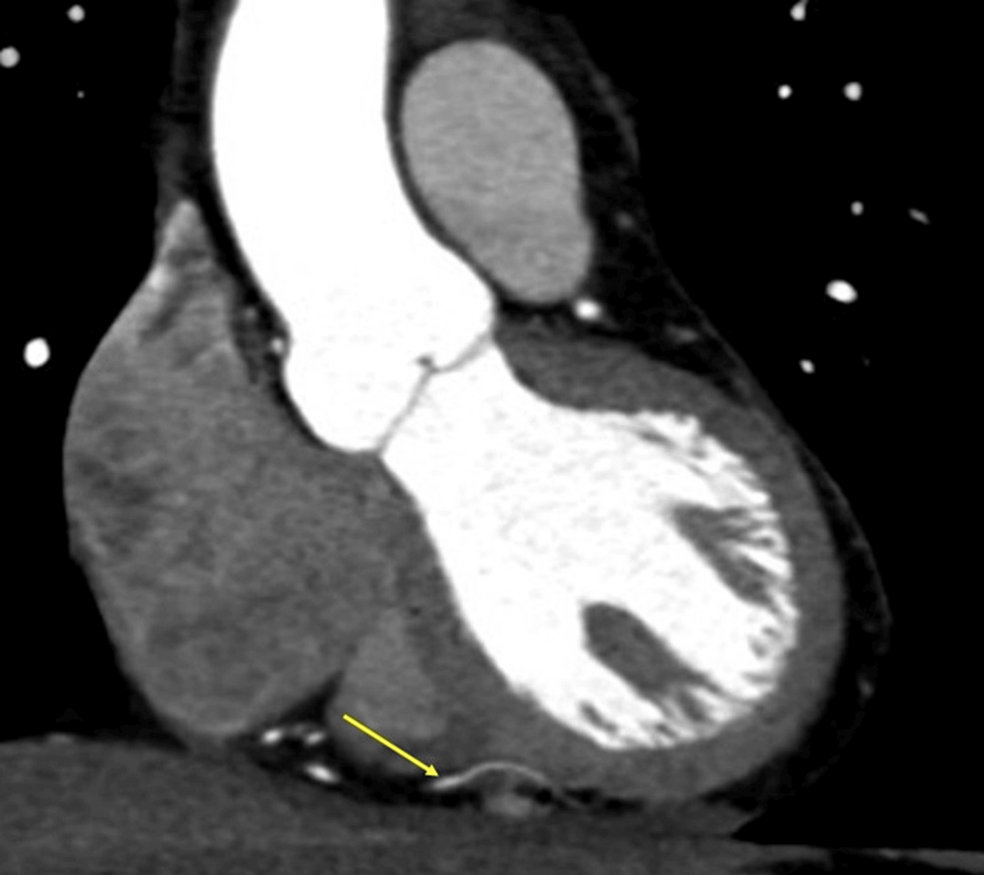
Superficial bridging. Superficial bridging in the posterolateral branch of the right coronary artery (yellow arrow) without luminal stenosis in the bridging segment. Coronal reformatted image.

**Figure 8:**
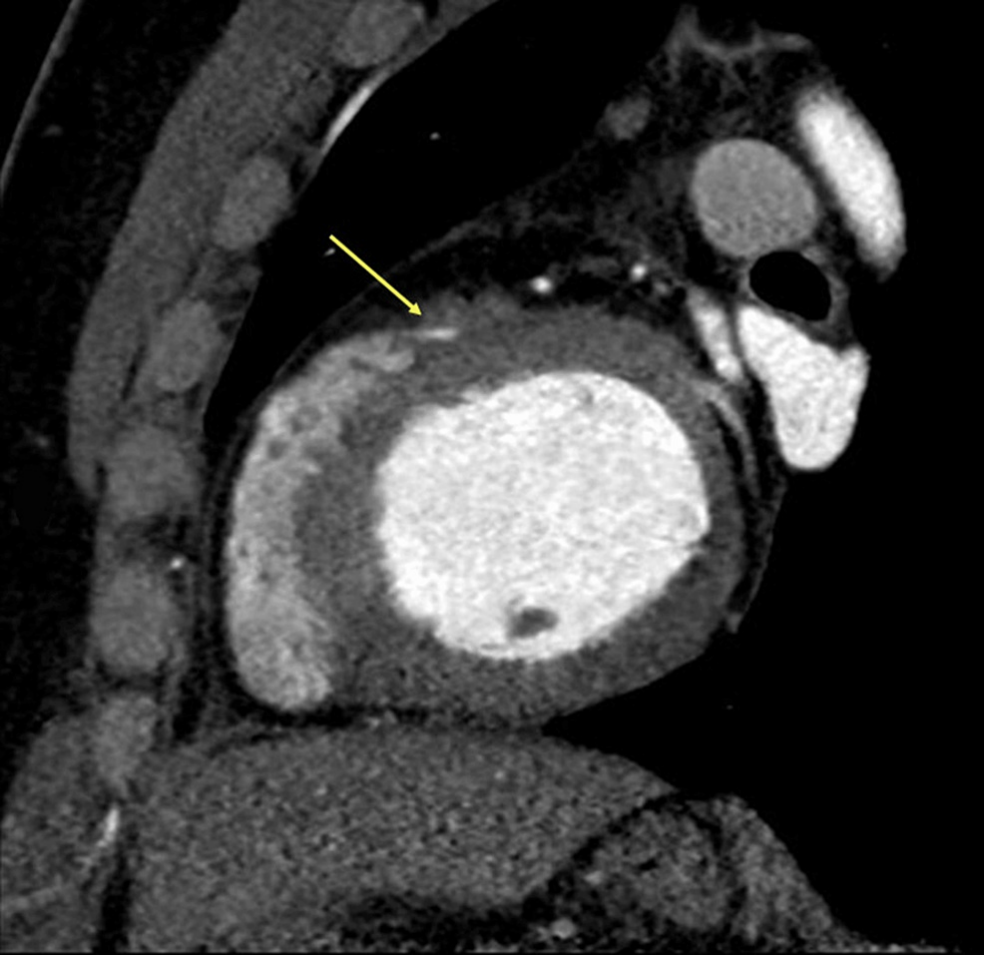
Deep bridging. Deep bridging in the mid-left anterior descending artery without luminal stenosis in the bridging segment (yellow arrow). Oblique sagittal image.

**Figure 9:**
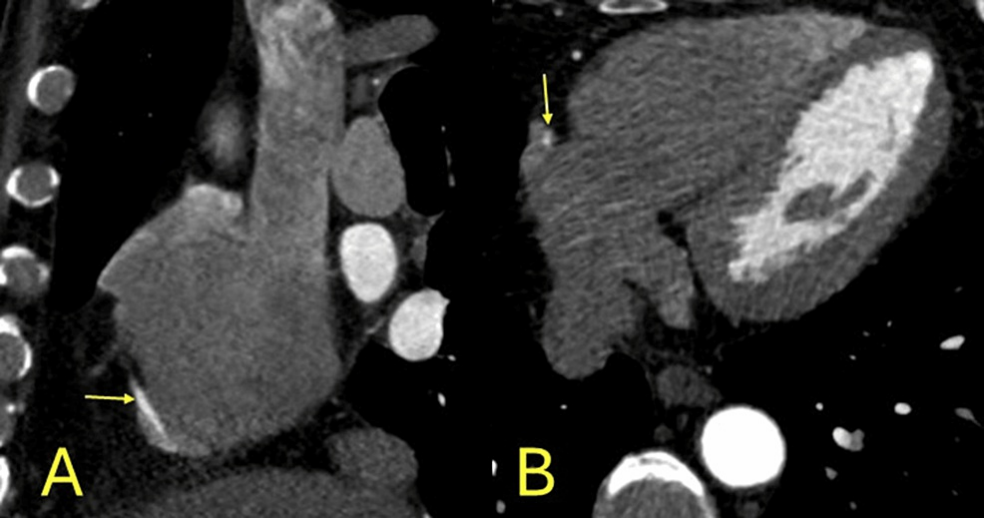
Deep bridging. Deep myocardial bridging of the distal right coronary artery without luminal stenosis in the bridging segment (yellow arrow). A: sagittal, reformatted, B: axial images.

We noted 26 intrinsic anomalies, including 25 counts of bridging and one count of ectasia or coronaries. The bridging cases were divided into 21 superficial (12 LAD and nine RCA) (Figures 6, 7) and four deep (two LAD and two RCA) (Figures 8, 9).

Incidental extra coronary abnormalities

**Figure 10:**
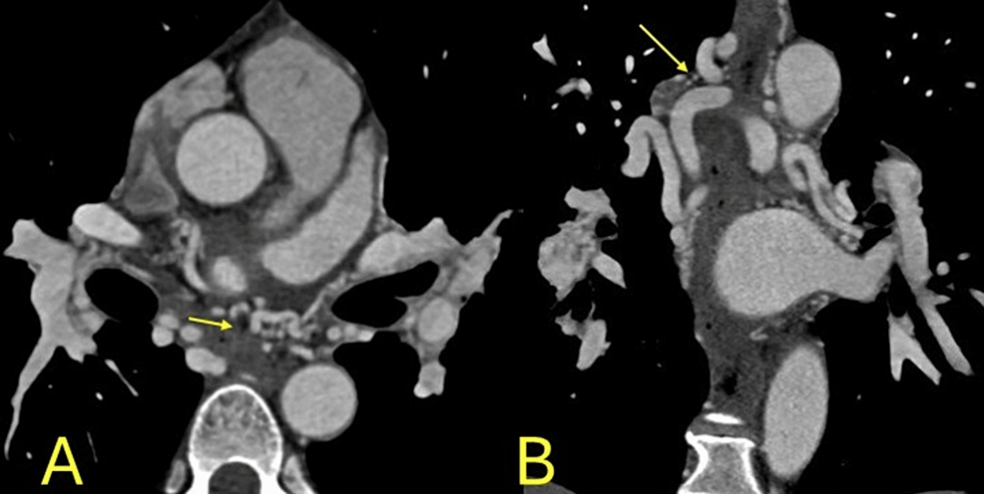
Collateral vessels. Collateral vessels were found in the mediastinum as an incidental finding of an unknown cause. Coronary arteries were found to be ectatic. The patient was lost to follow-up. A: axial and B: coronal reformatted images show vascular malformation with collateral vessels (yellow arrows).

There were five cases of incidental extracoronary abnormalities noted, i.e., total anomalous pulmonary venous drainage (TAPVD), sarcoidosis, distal oesophagal leiomyoma, mediastinal arteriovenous (AV) fistula, and vasculitis noted in mediastinal vessels (Figure 10). The case with vasculitis showed additional superficial myocardial bridging (MB); one case with AV fistula also showed coronary ectasia; and one case showed both superficial MB of RCA and deep MB of LAD.

## Discussion

The distribution of CAA variants presents an important insight into clinical practice and guides future clinical assessment focus. The study highlights the high proportion of MB, with 22 cases of superficial bridging and one case of deep bridging. Notably, the study identified four cases each of anomalous origin of the RCA and anomalies of origin and course of the LCx artery, two cases of absent LCx artery, and a singular instance of an absent left main coronary artery with separate ostia for the RCA and LCx. Additionally, the presence of a coronary artery fistula, a rare but significant anomaly, was documented in one patient. Alongside, five extracardiac causes, including TAPVD, gastrointestinal stromal tumour, sarcoidosis, and arteritis, further illustrate the diverse aetiology of observed anomalies.

Prevalence

Studies indicate that CAAs affect approximately 1% of the general population and 0.3% of all patients undergoing autopsy, with prevalence rates ranging from 0.3% to 5.6% in patients undergoing coronary angiography [7,12,13] The prevalence of anomalous coronary arteries is higher for the specific subgroups of patients, for example, with tetralogy of Fallot (ToF). A meta-analysis of 28 studies and 6,965 patients revealed that 6% of patients had ToF, of which 72% crossed the right ventricular outflow tract and were essential during procedure planning [14].

Clinical implications

Although congenital CAAs are relatively uncommon, they constitute the second most common cause of sudden cardiac death among young athletes, with a prevalence ranging from 5% to 17% [15,16]. These anomalies can result in severe myocardial ischemia, leading to symptoms such as chest pain, arrhythmias, and sudden cardiac death.

Multiple studies have looked into surgery for anomalous vessels in children and young adults (below 30 years). A systematic review and meta-analysis of 13 publications and 384 patients with anomalous aortic origin of a coronary artery revealed that the predominant surgical approach was unroofing of the intramural segment, which was utilised in 92% of cases. The pooled early and late mortality rates of 0% and 0.1%, respectively, and the instances of reoperation for aortic regurgitation were rare. The findings suggest that surgical management of anomalous aortic origin of coronary arteries can yield excellent results in pediatric patients; however, concerns regarding the long-term durability of surgery persist [17].

In the elderly population, the likelihood of sudden death attributed to anomalous vessels is considerably lower, as symptoms typically manifest earlier in life rather than later [13]. The underlying mechanism of death often involves hypoperfusion secondary to vessel compression, which may occur due to factors such as acute angles of origin, intramural course, or compression between adjacent vessels. These factors, exacerbated during strenuous exercise, disproportionately increase the incidence of sudden death among athletes.

Notably, the presence of CAAs increases the risk associated with routine procedures, underscoring the importance of prior knowledge of their presence.

When evaluating these subtypes of CAAs, it was initially believed that the presence of a left coronary artery arising from the right sinus posed a higher risk of sudden cardiac death. However, there is growing acknowledgement that a dominant RCA originating from the left sinus of Valsalva, exhibiting high-risk anatomic features, may present an equivalent risk [7,13,18-20].

The American College of Cardiology and American Heart Association guidelines propose the following three criteria for Class I indications for surgical intervention: (1) the presence of an anomalous left main coronary artery coursing between the aorta and pulmonary artery (PA); (2) ischemia resulting from coronary compression, either between the great arteries or in an intramural manner; and (3) an anomalous RCA origin between the aorta and PA, accompanied by evidence of ischemia [13,21].

Although coronary CT is the gold standard for evaluating coronary arteries, MR coronary evaluation can demonstrate well their origin and proximal course. It can also show associated myocardial involvement, such as ischemia or cardiomyopathy. However, the availability of MRI and expertise may not be readily available. It is also more expensive and may suffer from motion artefacts due to procedure time. It can, however, be undertaken if radiation exposure is a consideration [22].

Limitations

Limitations of our single-centre, retrospective analysis include potential selection bias of hospitalised patients due to the study’s retrospective design, limited generalizability of findings to broader populations, and inability to establish a link with the clinical symptoms due to the imaging basis of the study.

Future directions

Future studies should strive to elucidate the pathophysiological mechanisms that associate specific types of CAAs with myocardial ischemia, alongside evaluating their actual influence on the personal risk of critical incidents. The infrequent nature of these anomalies, combined with their clinical and phenotypic diversity and ethical constraints, might challenge setting up prospective studies in this domain. However, international collaborations and the development of multicentre registries might offer a pathway to address these [4].

## Conclusions

The aim of this study was to describe common variants of CAAs with the aid of non-invasive medical images (CTA) based on the review of all cardiac imaging from a single hospital. By documenting and analysing the characteristics of CAAs, it contributes valuable insights into the prevalence and imaging features of these anomalies, enhancing the body of knowledge necessary for improving patient outcomes in cardiovascular care.
